# Prevalence and Impact of Contraceptive Misconceptions Among Married Women in Al-Ahsa, Saudi Arabia

**DOI:** 10.3390/healthcare13243256

**Published:** 2025-12-12

**Authors:** Rasha Ahmed Fouad, Ghadah Abdulaziz AlShaikh-Mubarak, Fai Fahad Alruwaished, Noura Yousef Alrasheed, Raghad Abdullah Alessad, Rawan Nabeel Alabdullah, Norah Adel Alali, Latifah Saleh Aljawf

**Affiliations:** 1 College of Nursing, King Saud Bin Abdulaziz University for Health Sciences, Al-Ahsa 36361, Saudi Arabia; shikhmubarakg@ksau-hs.edu.sa (G.A.A.-M.); nourayousef929@gmail.com (N.Y.A.);; 2 King Abdullah International Medical Research Center, Al-Ahsa 31982, Saudi Arabia; 3 Gerontological Nursing Department, Faculty of Nursing, Alexandria University, Alexandria 21511, Egypt

**Keywords:** contraceptive use, misconceptions, reproductive health, women’s health, Saudi Arabia

## Abstract

Background: Family planning is essential for improving maternal and child health. However, misconceptions and cultural beliefs remain significant barriers to contraceptive use in many conservative societies. Purpose: This study aimed to assess the prevalence of contraceptive misconceptions and examine their impact on contraceptive use among married women in Al-Ahsa, Saudi Arabia. Methods: A cross-sectional study was conducted from January to April 2023, enrolling 379 married women aged 18–49 years from primary healthcare centers in Al-Ahsa. A structured, validated questionnaire was used to collect data on sociodemographic characteristics, contraceptive practices, and the prevalence of common misconceptions. Statistical tests were performed for data analysis using SPSS version 26. Results: Misconceptions were highly prevalent, with 94.7% believing contraceptives affect mood, 92.3% linking them to hormonal disorders, and 63.3% to impaired future pregnancies. Conclusions: The findings indicate that widespread misconceptions about contraceptives persist among married women in Al-Ahsa. These beliefs significantly limit the optimal use of family planning methods, despite relatively high educational attainment in the population. The study highlights an urgent need for targeted, culturally sensitive interventions to correct misinformation and improve women’s reproductive health outcomes.

## 1. Introduction

Family planning is a vital component of reproductive healthcare, significantly contributing to the improvement of maternal and child health outcomes. The effective use of contraceptives is linked to lower rates of maternal and infant mortality, a reduction in unintended pregnancies, and the broader empowerment of women through increased autonomy and socioeconomic participation [1]. Despite global advances in the accessibility and awareness of contraception, significant barriers to its use remain in many regions, often rooted in sociocultural resistance, religious interpretations, and pervasive misinformation [2].

In Saudi Arabia, reproductive health behaviors are deeply shaped by cultural norms, religious values, and traditional gender roles that influence perceptions of contraception and family planning. Although national initiatives have expanded access to reproductive healthcare services, the uptake of modern contraceptive methods remains disproportionately low relative to general awareness levels [3]. Modern contraceptive methods, including oral pills, intrauterine devices (IUDs), and injectables, are widely available free of charge at Ministry of Health primary healthcare centers, and barrier methods such as condoms can be purchased over the counter. Despite this accessibility, national surveys indicate that only approximately 30–35% of Saudi women of reproductive age currently use modern contraceptive methods [1].

In many households, reproductive decisions are influenced by husbands or extended family members, limiting women’s autonomy in method selection. Furthermore, religious interpretations and social taboos surrounding contraceptive use contribute to limited open dialogue and persistent misinformation, particularly in conservative regions. This paradox between accessibility and sociocultural restriction underscores the need for culturally sensitive strategies to improve contraceptive utilization in Saudi Arabia [2].

Persistent myths and misinformation, such as fears of infertility, hormonal disturbances, and long-term health complications, continue to deter women from using contraceptives, despite their proven safety and free availability at primary healthcare centers [4]. Furthermore, the social sensitivity surrounding discussions of family planning, coupled with male-dominated decision-making, limits open communication about reproductive health issues. These sociocultural constraints, compounded by inadequate provider-initiated counseling, perpetuate misconceptions and hinder the optimal utilization of family planning methods [5,6,7].

Regional studies have identified education, spousal influence, and religious interpretation as key determinants of contraceptive practices among women in the Kingdom [8,9,10]. However, limited research has examined how these factors interact with misinformation to shape contraceptive behaviors within the Eastern Province, particularly in Al-Ahsa. Understanding these dynamics within the local sociocultural context is critical for developing culturally responsive strategies that promote informed decision-making and enhance reproductive health outcomes.

Accordingly, the present study aimed to assess the prevalence and nature of contraceptive misconceptions among married women in Al-Ahsa, Saudi Arabia, and to examine their association with sociodemographic characteristics to inform evidence-based educational interventions.

This study was therefore conducted to assess the prevalence and specific nature of misconceptions regarding contraceptive use among married women in Al-Ahsa. By identifying the most common myths and their association with sociodemographic factors, the findings of this research aim to provide valuable, evidence-based insights to inform the development of culturally sensitive health education strategies and strengthen family planning interventions tailored to this population’s needs. The Al-Ahsa region was selected due to its representative nature as one of the largest and most socio-culturally diverse governorates in the Eastern Province, encompassing a mixed population across urban and rural communities with varying educational and socioeconomic levels. This context makes it a suitable setting for examining the influence of cultural and contextual factors on contraceptive beliefs and practices.

## 2. Materials and Methods

### 2.1. Design and Setting

This was a cross-sectional, observational study conducted from January to April 2023. The research was carried out in five randomly selected primary healthcare centers in Al-Ahsa, Eastern Province, Saudi Arabia. These locations were chosen to ensure a representative sample from both urban and rural areas of the region. 

### 2.2. Population and Eligibility

The study population included married women between 18 and 49 years old who were attending the selected healthcare centers during the study period. We excluded women who were pregnant, unmarried, had a history of infertility, or had known cognitive impairments. All participants provided informed written consent before enrollment.

### 2.3. Sample Size and Sampling Procedure

The minimum required sample size was calculated as 379 participants. Sample size calculations were conducted using G*Power (version 3.1.9.7; Heinrich-Heine-University Düsseldorf, Düsseldorf, Germany). A multi-stage random sampling technique was used to select participants. Initially, five PHCs were randomly selected from a comprehensive list of primary healthcare facilities across the Al-Ahsa region. Subsequently, within each selected PHC, systematic random sampling was utilized. Every third eligible woman presenting during routine clinic hours was approached and invited to participate in the study until the target sample size was reached.

### 2.4. Data Collection

Data were collected using a structured, interviewer-administered questionnaire provided in Arabic. The development of this instrument was necessary because no standardized, culturally adapted Arabic tool existed in the literature to comprehensively assess the specific misconceptions and beliefs about contraceptive use among Saudi women; existing tools primarily focus on knowledge and attitudes. Therefore, the questionnaire items were formulated based on an extensive literature review [11,12,13,14,15] and expert consultation to ensure relevance to the specific Saudi cultural context.

The questionnaire included a section on Misconceptions related to each method. For the purpose of this study, a contraceptive misconception was defined as a belief about a contraceptive method that constitutes misinformation or a misperception not supported by scientific evidence.

The final questionnaire was designed to gather information across three key areas:

**Sociodemographic and Reproductive Characteristics**: This section included age, education level, parity, household income, and history of previous contraceptive use.

**Contraceptive Methods and Misconceptions**: Participants were asked about current and previous use of any contraceptive methods, including oral pills, intrauterine devices (IUDs), injectable, and barrier methods such as condoms. Misconceptions related to each method were then assessed, though oral contraceptive use predominated among respondents. This section was developed by the research team following a review of relevant literature [11,12,13,14,15] to assess common myths and beliefs. Key areas explored included fears of infertility, hormonal disturbances, weight gain/obesity, and potential complications with future pregnancy.

To ensure accuracy, the questionnaire underwent forward and backward translation. It was then pilot-tested with 30 women who were excluded from the final study analysis. The resulting internal consistency of the instrument was confirmed to be strong (Cronbach’s α = 0.82).

**Procedure**

Data collection was performed by trained female healthcare professionals in a private setting to protect participant confidentiality. Visual aids were employed during the administration to help clarify any complex medical terminology; ensuring participants fully understood the questionnaire items.

### 2.5. Ethical Approval

Ethical approval for the study was granted by the King Abdullah International Medical Research Ethics Committee (IRB/2219/23, 3 September 2023). All procedures were in full compliance with the Declaration of Helsinki. Participants were fully informed about the study’s objectives. Written informed consent was obtained before participation. Data were anonymized and securely stored. Participants were assured of their right to withdraw at any stage without any penalty.

### 2.6. Statistical Analysis

All data were analyzed using IBM SPSS Statistics, Version 26.0 (IBM Corp., Armonk, NY, USA). Descriptive statistics, including means, standard deviations (SDs), frequencies, and percentages, were calculated to summarize participant characteristics and study outcomes.

Associations between categorical variables were assessed using the Chi-square (χ^2^) test. Differences in mean awareness scores across sociodemographic categories (e.g., age groups, educational levels) were examined using one-way analysis of variance (ANOVA). When significant differences were detected, post hoc comparisons were conducted using Tukey’s test. Statistical significance was set at a two-tailed *p* < 0.05. All tests were two-tailed with significance set at *p* < 0.05. Figure was created using Microsoft PowerPoint (Microsoft Corp., Redmond, WA, USA).

Differences between urban and rural participants were not analyzed as separate subgroups due to unequal sample sizes (urban, n = 326; rural, n = 53), which could reduce statistical power. Future studies with a more balanced representation are recommended to further explore potential regional variations.

## 3. Results

### 3.1. Socio-Demographic Characteristics

Table 1 details the socio-demographic characteristics of the 379 married women who participated in the study. The cohort was predominantly composed of women aged 35 years or older (65.2%), with only a small minority (12.4%) being younger than 25. Most participants were highly educated, with over two-thirds (66.2%) having completed a university education. The sample was nearly all Saudi nationals (97.4%), and the majority resided in urban areas (86.0%). A substantial portion of the women were housewives (56.7%), followed by clerical workers (28.0%). In terms of financial status, 62.0% reported having an “enough” income, while 34.3% reported “not enough.” The distribution of parity was relatively even, with 45.9% having one to three children and 44.3% having four to six, and 9.8% with seven or more children.

### 3.2. Contraceptive Use Patterns

Table 2 presents contraceptive use patterns among study participants. A majority of the women (66.5%) reported a history of contraceptive use, but only 23.2% were current users at the time of data collection. Among those who had used oral contraceptive pills, a significant proportion (43.5%) used them for less than one year, whereas 32.5% reported use for five years or more. A notable finding was that 45.4% of the women reported experiencing physical or psychological issues related to contraceptive use, compared to 54.6% who did not.

### 3.3. Contraceptive Misconception and Awareness

Table 3A shows the high prevalence of common misconceptions regarding contraceptive use. Misinformation was widespread, with nearly all participants believing that contraceptives affect psychological state or mood (94.7%) and cause hormonal disorders (92.3%). Other prevalent misconceptions included the belief that contraceptives cause obesity (79.2%), polycystic ovary syndrome (66.0%), and infertility (61.2%). Additionally, a majority of women believed that contraceptives could affect future pregnancies (63.3%) or were associated with prolonged menstrual bleeding (59.1%).

Table 3B presents the comparison of mean awareness scores across different socio-demographic groups. A one-way analysis of variance (ANOVA) was used to assess differences in mean scores among the three age categories. Although women aged 35 years or older had a slightly higher mean awareness score (7.24 ± 2.55) than those aged 25–34 years (6.80 ± 2.45) and those under 25 years (6.53 ± 2.59), this difference was not statistically significant (ANOVA, *p* = 0.126). Similarly, another one-way ANOVA test was applied to compare awareness scores across the five educational levels, and no significant association was observed (*p* = 0.420), despite illiterate women showing the highest mean score (9.00 ± 2.83).

### 3.4. Prevalence of Contraceptive Misconceptions

**Figure 1:**
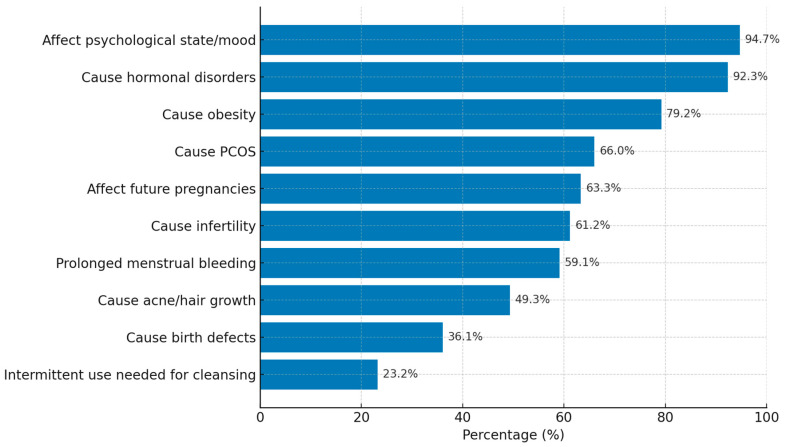
Top Misconceptions about Contraceptive Use among Married Women (n = 379).

Figure 1 illustrates the most frequently reported misconceptions about contraceptive use among married women in Al-Ahsa. As shown, misinformation was highly prevalent. The most dominant misconception was the belief that contraceptives affect a woman’s psychological state or mood (94.7%), closely followed by the belief that they cause hormonal disorders (92.3%) and obesity (79.2%). Misconceptions directly related to reproductive health were also common: 63.3% of participants believed that contraceptives could affect future pregnancies, and 61.2% believed they cause infertility. Other prevalent misconceptions included the beliefs that contraceptives because polycystic ovary syndrome (66.0%), prolonged menstrual bleeding (59.1%), and acne or excess hair growth (49.3%). Approximately one-third of the women believed contraceptives cause congenital birth defects (36.1%).

## 4. Discussion

To our knowledge, this is one of the first studies in Al-Ahsa, Saudi Arabia, to systematically investigate the impact of misconceptions on contraceptive behaviors among married women. This research offers locally relevant insight into the sociocultural determinants of family planning in this region. Our findings reveal a high prevalence of contraceptive misconceptions, even among women with high educational attainment and general health awareness. These misconceptions significantly influence women’s decisions and hinder optimal family planning practices. The observed patterns emphasize the critical need to interpret health behaviors within the local context, considering how cultural norms and socio-religious factors intersect with health education to shape family planning outcomes.

The sociodemographic profile of our participants predominantly urban, university-educated, and of reproductive age would typically be associated with higher contraceptive uptake. Previous studies in Saudi Arabia and other Middle Eastern countries have reported a positive correlation between higher education and contraceptive use [16,17]. However, our results showed that only 23.2% of women were current users, even though 66.5% had used contraceptives previously. This inconsistency suggests that education alone may not be enough to counter entrenched cultural myths, which remain a strong determinant of health behaviors. The divergence from earlier Saudi-based studies [16,18] highlights the influence of social discourse, family pressures, and misinformation that continue to override formal knowledge.

The dominance of oral contraceptive pills and intrauterine devices in our cohort is consistent with national and regional patterns [17,19]. Nevertheless, the relatively short duration of oral pill use and high discontinuation rates reported by many women reflect the experience of side effects, which are well-documented as common reasons for discontinuation in Saudi Arabia and globally [18,20]. Our findings that nearly half of the women reported adverse physical or psychological symptoms further reinforce the importance of individualized counseling and follow-up, as emphasized in previous literature [20].

A central finding of this study is the overwhelming presence of misconceptions regarding contraceptive use. The majority of participants believed that contraceptives cause hormonal disorders (92.3%), affect mood (94.7%), and contribute to obesity (79.2%) (as shown in Figure 1). Misbeliefs about infertility (61.2%) and impaired future pregnancies (63.3%) were also widespread. Misbeliefs about infertility (61.2%) and impaired future pregnancies (63.3%) were also widespread. These findings parallel those from South Africa and Uganda, where myths surrounding infertility and long-term health complications significantly discouraged contraceptive uptake [7,14,21]. Similarly, international evidence from South Asia and Sub-Saharan Africa demonstrates that anecdotal accounts and misinformation transmitted through social networks often outweigh medical advice [10,11,22].

Although oral contraceptive pills were the most frequently used method among participants, the reported misconceptions were not limited to this one method. Concerns have also been expressed regarding intrauterine devices (IUDs) and injectable methods, often citing fears of pain, infertility, or hormonal imbalance. Significantly, a few participants reported substituting hormonal methods with barrier methods such as condoms due to perceived side effects or negative beliefs. These findings collectively suggest that misinformation influences not only the initial decision to use contraception but also method choice, switching, and long-term continuity of effective family planning [7,10,14]. Therefore, culturally sensitive counseling must comprehensively address misconceptions across all available contraceptive options.

Although nearly 95% of women believed that contraceptives affect mood, this perception requires nuanced interpretation. A substantial proportion of participants reported experiencing mood-related symptoms personally. Rather than categorizing these entirely as misconceptions, this finding underscores the critical need for healthcare providers to acknowledge women’s lived experiences while offering balanced information regarding the physiological and psychological effects of hormonal methods. Evidence suggests that individual sensitivity to hormonal changes may contribute to such experiences, though systematic reviews show inconsistent findings regarding causality. The similarity of these patterns across diverse cultural contexts underscores that while the specific content of misconceptions may vary, their persistence is a global challenge in reproductive health [21,23].

However, unlike studies in other Saudi regions that demonstrated a clear association between education and knowledge [16,23], our study found no significant relationship between education or age and awareness levels. This suggests that in Al-Ahsa, even educated women remain susceptible to misinformation due to strong sociocultural influences and limited open discourse on reproductive health. The researcher interprets this divergence as indicative of the deeply embedded nature of these myths, where higher formal education cannot offset the weight of collective community beliefs. This interpretation is consistent with social network theories, which posit that peer and familial influence can be more powerful than factual information in shaping reproductive choices [24].

Another critical finding is the gap in healthcare provider engagement. Nearly half of the participants reported receiving no structured counseling, despite attending primary healthcare centers. This contrasts with evidence showing that effective provider counseling enhances satisfaction, continuation, and informed method selection [20]. The researcher interprets the problem as a systemic issue where family planning is available but underutilized due to insufficient integration of culturally sensitive counseling. Recent work from Saudi Arabia highlights the importance of tailoring health messages to cultural expectations, including digital and community-based approaches to counter persistent myths [25,26,27].

In summary, this study demonstrates that while contraceptive awareness exists, misconceptions remain the dominant barrier to uptake among Saudi women in Al-Ahsa. The similarities with international findings support the universality of misinformation as a barrier, while the differences, particularly the lack of an education effect, emphasize the distinct role of the sociocultural context in Saudi Arabia. These interpretations justify the urgent need for policy-level interventions that prioritize structured, culturally sensitive education strategies.

## 5. Implications for Practice

The findings of this study underscore an urgent need for enhanced, culturally sensitive reproductive health counseling in Saudi Arabia. Despite the high level of education among many participants, misconceptions about contraceptives were widespread and significantly influenced their perceptions and decisions.

The urgent need for enhanced, culturally sensitive counseling in Saudi Arabia extends beyond simply promoting contraceptive uptake. It is vital to ensure that women can make informed, autonomous choices among available methods and continue their chosen method safely and confidently. Whether a woman transitions from oral pills to barrier methods or seeks advice on long-term options, comprehensive counseling is essential to support the continuity of effective family planning.

To address this, healthcare providers should adopt individualized, client-centered counseling that directly confronts prevalent myths, such as those related to infertility, hormonal imbalances, and negative effects on future pregnancies. Training programs for primary healthcare staff are highly recommended to improve their capacity to provide accurate information and effectively counter misinformation. Furthermore, incorporating digital health platforms and community-based awareness campaigns could broaden outreach, especially for women with limited access to clinical healthcare. Given that misconceptions persisted even among university-educated women, culturally sensitive counseling must engage both educated and less-educated groups through context-relevant language and examples.

## 6. Limitations

The interpretation of the findings must be considered within the context of several limitations.

**Study Design and Scope**

The cross-sectional design of the study precludes the establishment of causal relationships between contraceptive misconceptions and patterns of contraceptive use. Furthermore, the study’s scope was geographically limited to married women attending primary healthcare centers in Al-Ahsa, which reflects the prevailing cultural norms regarding reproductive health in Saudi Arabia. Consequently, the results may not be generalizable to other populations, such as unmarried women, adolescents, or individuals in different regions of Saudi Arabia.

**Data Collection and Bias**

A key limitation is the reliance on self-reported data, which may be susceptible to recall bias regarding past behavior and social desirability bias, particularly concerning sensitive reproductive health topics due to cultural factors. Although the study utilized a large sample size and a systematic sampling approach to enhance validity within the target population, the sample included a limited number of participants from rural areas. This restriction prevented a detailed comparative analysis of contraceptive misconceptions between rural and urban populations within the Al-Ahsa region.

## 7. Conclusions and Recommendations

While contraceptive awareness among married women in Al-Ahsa is high, misconceptions remain pervasive, especially concerning infertility, hormonal imbalances, and adverse effects on future pregnancies. These beliefs contribute to the underutilization of contraceptive methods despite widespread knowledge.

To improve reproductive health outcomes, comprehensive strategies are necessary. These should include structured, provider-led counseling, the development of culturally sensitive educational materials, and the use of digital health interventions to effectively dispel myths. Policymakers and healthcare leaders should prioritize reproductive health education in both primary care and community settings, ensuring that accurate, accessible, and culturally appropriate information reaches women of all backgrounds. Future research should include more diverse populations, such as unmarried and adolescent women, to provide a more holistic understanding of reproductive health needs throughout Saudi society.

## Figures and Tables

**Table 1:** Socio-Demographic Characteristics of Married Women in Al-Ahsa, Saudi Arabia (n = 379).

Characteristic	n	%
Age (years)		
<25	47	12.4
25–34	85	22.4
≥35	247	65.2
Educational level		
Illiterate	2	0.5
Read and write	4	1.1
Primary education	12	3.2
Secondary education	110	29.0
University education	251	66.2
Nationality		
Saudi	369	97.4
Non-Saudi	10	2.6
Residence		
Urban	326	86.0
Rural	53	14.0
Occupation		
Housewife	215	56.7
Clerical worker	106	28.0
Craft worker	8	2.1
Others	50	13.2
Income		
Not enough	130	34.3
Enough	235	62.0
More than enough	14	3.7
Number of children		
1–3	174	45.9
4–6	168	44.3
≥7	37	9.8

**Table 2:** Contraceptive Use Patterns among Married Women in Al-Ahsa, Saudi Arabia (n = 379).

Variable	n	%
Ever used contraceptives		
Yes	252	66.5
No	127	33.5
Currently using contraceptives		
Yes	88	23.2
No	291	76.8
Duration of oral pill use (among users)		
<1 year	165	43.5
1–<3 years	56	14.8
3–<5 years	35	9.2
≥5 years	123	32.5
Reported physical or psychological problems		
Yes	172	45.4
No	207	54.6

**Table 3:** Misconceptions about Contraceptive Use and Awareness Scores by Socio-Demographic Characteristics.

**A. Common Misconceptions Reported (n = 379)**		
Misconception	Yes	(%)
Contraceptive pills cause infertility	232	(61.2)
Contraceptive pills cause obesity	300	(79.2)
Contraceptives cause hormonal disorders	350	(92.3)
Contraceptives affect psychological state/mood	359	(94.7)
Contraceptives cause PCOS	250	(66.0)
Contraceptives cause prolonged menstrual bleeding	224	(59.1)
Contraceptives cause acne/hair growth	187	(49.3)
Contraceptives cause birth defects	137	(36.1)
Intermittent use is necessary for cleansing	88	(23.2)
Contraceptives affect future pregnancies	240	(63.3)
**B. Awareness Score Differences by Socio-Demographic Factors**		
Variable	Mean ± SD	*p*-value
Age < 25	6.53 ± 2.59	
25–34	6.80 ± 2.45	0.126
≥35	7.24 ± 2.55	
Education		
Illiterate	9.00 ± 2.83	
Read & Write	7.75 ± 2.06	
Primary	7.42 ± 2.61	0.420
Secondary	6.73 ± 2.65	
University	7.15 ± 2.49	

Note: Descriptive statistics (frequencies and percentages) were used to summarize the prevalence of misconceptions (Part A). For group comparisons of mean awareness scores (Part B), one-way analysis of variance (ANOVA) was performed for variables with more than two categories (e.g., age and education). A two-tailed *p*-value < 0.05 was considered statistically significant.

## Data Availability

The datasets generated and analyzed during the current study are available from the corresponding author upon reasonable request. To ensure participant confidentiality, data will be shared in a de-identified format. All data and materials were handled and stored in a secure manner throughout the study.
